# Genome stabilization by RAD51‐stimulatory compound 1 enhances efficiency of somatic cell nuclear transfer‐mediated reprogramming and full‐term development of cloned mouse embryos

**DOI:** 10.1111/cpr.13059

**Published:** 2021-05-21

**Authors:** Ah Reum Lee, Ji‐Hoon Park, Sung Han Shim, Kwonho Hong, Hyeonwoo La, Kyung‐Soon Park, Dong Ryul Lee

**Affiliations:** ^1^ Department of Biomedical Science CHA University Seongnam Gyunggi‐do Korea; ^2^ CHA Advanced Research Institute CHA University Seongnam Gyunggi‐do Korea; ^3^ Department of Stem Cell and Regenerative Biology Konkuk University Gwangjin‐gu Seoul Korea

**Keywords:** homologous recombination repair, *Rad51*, reprogramming, RS‐1, somatic cell nuclear transfer

## Abstract

**Objectives:**

The genetic instability and DNA damage arise during transcription factor‐mediated reprogramming of somatic cells, and its efficiency may be reduced due to abnormal chromatin remodelling. The efficiency in somatic cell nuclear transfer (SCNT)‐mediated reprogramming is also very low, and it is caused by development arrest of most reconstituted embryos.

**Materials and Methods:**

Whether the repair of genetic instability or double‐strand breaks (DSBs) during SCNT reprogramming may play an important role in embryonic development, we observed and analysed the effect of *Rad 51*, a key modulator of DNA damage response (DDR) in SCNT‐derived embryos.

**Results:**

Here, we observed that the activity of *Rad 51* is lower in SCNT eggs than in conventional IVF and found a significantly lower level of DSBs in SCNT embryos during reprogramming. To address this difference, supplementation with RS‐1, an activator of *Rad51*, during the activation of SCNT embryos can increase RAD51 expression and DSB foci and thereby increased the efficiency of SCNT reprogramming. Through subsequent single‐cell RNA‐seq analysis, we observed the reactivation of a large number of genes that were not expressed in SCNT‐2‐cell embryos by the upregulation of DDR, which may be related to overcoming the developmental block. Additionally, there may be an independent pathway involving histone demethylase that can reduce reprograming‐resistance regions.

**Conclusions:**

This technology can contribute to the production of comparable cell sources for regenerative medicine.

## Graphical abstract

We determined the effect of upregulation of Rad51 homologous 1 (RAD51) by treatment with RAD51‐stimulatory compound 1 (RS‐1) on the role of DSB repair during somatic cell nuclear transfer (SCNT) reprogramming and the improvement of embryonic development by reducing DNA damage. Therefore, supplementation with RS‐1 during reprogramming can recover RAD51 activity and improve the derivation efficiency of PSCs, and would be an efficient, safe and simple protocol for improving the efficacy of SCNT technology.

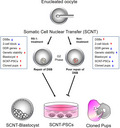

## 1 INTRODUCTION

Mammalian oocytes endow terminally differentiated somatic cells with totipotency through the reprogramming that occurs somatic cell nuclear transfer (SCNT), which has resulted in the production of genetically identical animals of more than 20 species.^1^, ^2^ Moreover, SCNT can be applied to therapeutic cloning through the generation of immunocompatible pluripotent stem cells (PSCs) from patient‐derived somatic cells, which makes SCNT a promising technology for in vitro disease modelling and cell transplantation.^3^ Various efforts have been undertaken to comprehensively recognize the pivotal molecular factors affecting successful generation of mammalian SCNT‐cloned embryos, conceptuses and offspring. These factors determine among others (a) the provenance of nuclear donor cells,^4^, ^5^, ^6^, ^7^ (b) quality of nuclear recipient oocytes related to their meiotic, epigenomic and cytoplasmic maturity status.^8^, ^9^, ^10^ The aforementioned factors also determine (c) epigenetic reprogrammability of donor cell nuclear genome in SCNT‐derived oocytes and resultant embryos,^11^, ^12^, ^13^, ^14^, ^15^ (d) intergenomic crosstalk between nuclear and mitochondrial compartments in SCNT‐derived oocytes and cloned embryos^16^, ^17^, ^18^, ^19^, ^20^ and (e) the incidence of apoptosis‐ or autophagy‐dependent events in the ex vivo‐expanded nuclear donor cells and cultured cloned embryos.^21^, ^22^, ^23^ Although SCNT presents tremendous potential for practical application in various areas, there are technical limitations that cause poor embryonic development, which has been largely attributed to the abnormal epigenetic status of the transplanted nuclei derived from somatic cells.^24^ In fact, the success rate of SCNT is mainly determined by the efficiency with which enucleated oocytes reprogramme the epigenetic identity of donor somatic nuclei before the onset of zygotic genome activation (ZGA), which occurs at the 2‐cell stage in mice and the 8‐cell stage in humans.^25^, ^26^, ^27^ In mice, the treatment of SCNT‐derived embryos with histone deacetylase inhibitors such as TSA significantly improves their development and epigenetic status.^28^ Furthermore, the application of another histone‐modifying enzyme, lysine‐specific demethylase 4D (KDM4D), for the reprogramming of somatic nuclei overcomes epigenetic barriers to embryo development at the 2‐cell stage through the regulation of the H3K9 methylation of donor nuclei.^25^ Additionally, the overexpression of the H3K27me3‐specific demethylase KDM6A, but not KDM6B, improves the efficiency of mouse SCNT.^29^ Recently, the derivation rate of human SCNT‐PSCs has been greatly increased by the injection of *KDM4A* mRNA to reduce H3K9me3 activity, but this methodology still does not seem to be as efficient as possible.^27^

Another mechanism that is highly likely to be associated with SCNT efficiency is genome stability. In another type of reprogramming procedure, the generation of induced pluripotent stem cells (iPSCs) is negatively related to the activation of TP53 by damaged DNA and the introduction of transcription factors (TFs) increases the phosphorylated form of H2AX (γH2AX) foci, which are markers of DNA double‐ and single‐stranded breaks.^30^, ^31^ Additionally, it was recently reported that significant genomic DNA breaks occur when the somatic cell nucleus is remodelled through chromosome condensation prior to entry into S‐phase.^32^ Similarly, γH2AX foci are detected at the time of paternal DNA demethylation,^33^ suggesting that genome stability is challenged by DNA breakage during zygotic reprogramming. Given the potential for genetic instability to impede embryonic development after SCNT, it can be postulated that increasing genome stability is another approach for enhancing the reprogramming efficiency of SCNT.

Rad51 homologous 1 (RAD51) is a DNA‐binding protein that has pleiotropic functions in maintaining genome stability and a regulating protein to control the DNA damage response (DDR), homologous recombination, DNA replication and repair.^34^ In fact, cells with spontaneous DNA damage display high expression of *rad51*, which is induced by multiple types of genotoxic stress.^35^ Furthermore, during the development of porcine parthenotes, inhibiting RAD51 induces apoptosis, reactive oxygen species accumulation, an abnormal mitochondrial distribution, delayed division during development and inhibition of development to the blastocyst stage.^36^ In 2017, Chia et al.^32^ found that genetic instability marked by frequent chromosome segregation errors and DNA double‐strand breaks (DSBs) arose prior to transcriptional activity during human and mouse SCNT reprogramming, and the absence of the repair this damage resulted in delayed DNA replication and severely abnormal mitosis. On the basis of these results, we hypothesized that the repair of genetic instability or DSBs during SCNT reprogramming plays an important role in embryonic development. In the present study, we found that increased RAD51 activity induced by supplementation with RAD51‐stimulatory compound 1 (RS‐1), a homology‐directed repair (HDR) enhancer, increases genomic remodelling during the early phase of mouse SCNT and subsequently enhances reprogramming efficiency. This approach may contribute to the reprogramming of somatic cells independent of previous epigenetic modifications, and the use of both approaches as a combined protocol will have a synergistic effect in improving the efficiency of reprogramming by SCNT.

## 2 MATERIALS AND METHODS

### 2.1 Mice

Eight‐ to 10‐week‐old female B6D2F1 mice (Orient‐Bio Inc) were used both for the collection of recipient oocytes and as SCNT donors. Eight‐ to 10‐week‐old female ICR mice were used as the foster mothers in embryo transfer. To induce pseudopregnancy, these mice were mated with vasectomized male mice of the same strain. The protocols for the use of animals in these studies were approved by the Institutional Animal Care and Use Committee (IACUC) of CHA University (Project No. IACUC‐170119, IACUC‐180053, IACUC‐190070), and all experiments were carried out in accordance with the approved protocols.

### 2.2 Preparation of oocytes and nuclear donor cells

Both oocytes and cumulus cells were prepared by the superovulation of 8‐ to 10‐week‐old B6D2F1 female mice. Superovulation was induced by sequential injection 5 IU of pregnant mare serum gonadotropin (Sigma‐Aldrich), followed by 5 IU of human chorionic gonadotropin (hCG, Sigma‐Aldrich) with an interval of 48 hours. Cumulus‐oocyte complexes (COCs) were collected from oviducts in M2 medium (Sigma‐Aldrich) at 14 hours after hCG injection and were treated with M2 containing 0.1% bovine testicular hyaluronidase (Sigma‐Aldrich) to obtain dissociated cumulus cells and oocytes. The cumulus‐free oocytes were then cultured in potassium simplex optimized medium (KSOM; Millipore) at 37°C under 5% CO_2_ until further use. Dispersed cumulus cells were dissociated by hyaluronidase treatment, diluted in M2 medium and collected. The pellet was then resuspended in a small volume of polyvinylpyrrolidone (PVP) in M2 medium in a manipulator chamber.

### 2.3 Preparation of *Kdm4a* mRNA

In vitro transcription was performed as described previously.^27^ In brief, full‐length mouse *Kdm4a/Jhdm3a* cDNA was cloned into a pcDNA3.1 plasmid containing poly(A)83 at the 3′ end of the cloning site by using an In‐Fusion Kit (#638909, Clonetech). Messenger RNA was synthesized from the linearized template plasmids by in vitro transcription using a mMESSAGE mMACHINE T7 Ultra Kit (#AM1345, Life Technologies). The synthesized mRNA was dissolved in nuclease‐free water. The concentration of mRNA was measured using a NanoDrop ND‐1000 spectrophotometer (NanoDrop Technologies); aliquots of mRNA were stored at −80°C until use.

### 2.4 Mouse SCNT procedure and RS‐1 treatment

**FIGURE 1:**
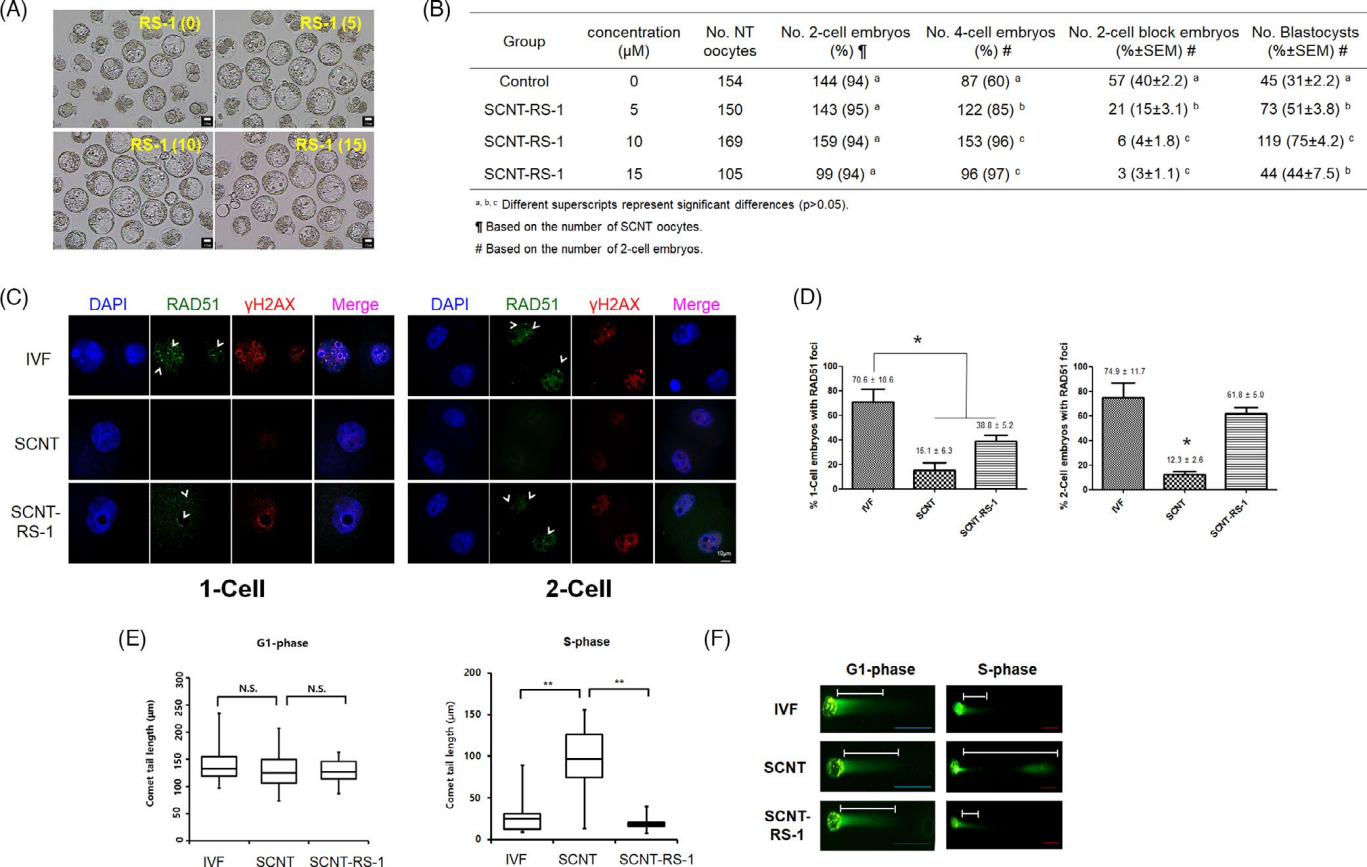
Treatment with rad51‐stimulatory compound 1 (RS‐1) improved the embryonic development of SCNT embryos and reduced DNA double‐strand breaks (DSBs). A, Blastocyst formation in SCNT embryos treated with RS‐1 at different concentrations. Scale bar, 10 µm. B, Effect of RS‐1 treatment at different concentration on the developmental potential of cloned embryos. C, Immunostaining of RAD51 and γH2AX foci for detection of DNA DSBs and DNA repair activity in the IVF, SCNT, and SCNT‐RS‐1 groups at the 1‐cell and 2‐cell stages. D, The percentage of embryos with RAD51 foci at the 1‐cell (left) and 2‐cell stages (right) cloned embryos in the IVF, SCNT, and SCNT‐RS‐1 groups (^*^ *P* < .05). This experiment was repeated more than three times and the number of embryos was more than 10 (per each group). E, DNA breaks with the average comet tail lengths in G1‐phase (5 h) and S‐phase (8 h) 1‐cell stage embryos from the IVF, SCNT, and SCNT‐RS‐1 groups determined through the comet assay. This parameter essentially represents the product of the percentage of the total DNA in the tail and the distance between the centres of mass of the head and tail regions (^**^ *P* < .01). F, Representative photographs of the comet assay results at G1‐phase and S‐phase in the IVF, SCNT, and SCNT‐RS‐1 groups

All MII stage oocytes with distinct first polar bodies were enucleated in M2 medium containing 5 µg/mL cytochalasin B. For nuclear transfer, cumulus cells were injected into enucleated oocytes in M2 medium using a PIEZO‐driven micromanipulator (Primetech). After nuclear transfer, the reconstructed oocytes were incubated in KSOM medium for 1‐2 hours before activation. Activation was performed by incubation in M16 (Millipore) medium containing 10 mmol/L SrCl_2_, 2 mmol/L EGTA, and 5 µg/mL cytochalasin B for 6 hours, and then, the oocytes were cultured in KSOM in a humidified atmosphere of 5% CO_2_ at 37°C. During activation, the treated groups were supplemented with RAD51‐stimulatory compound 1 (RS‐1; R9782, Sigma‐Aldrich), which was continued for 22 hours after activation, until the 2‐cell stage. In preliminary experiment, embryonic development was monitored according to treating time for 22 (the time of first cleavage), 48 and 72 hours, and then, treatment of RS‐1 for 22 hours was chosen because of high rate of blastocyst formation (Data not shown). The concentration of RS‐1 was chosen from test comparing the results of treatment with or without 5 µmol/L, 10 µmol/L and 15 µmol/L RS‐1 (Figure 1A,B). The embryonic development of cloned embryos was assessed for 5 days (120 hours) after activation.

### 2.5 In vitro fertilization and embryo culture

Sperm masses were collected from the cauda epididymis and placed in drops of incubated HTF (Millipore) medium covered with mineral oil. The spermatozoa were capacitated for 1 hour under 5% CO_2_ at 37°C before being used for insemination. As an experimental control, MII oocytes containing COCs were collected from B6D2F1 female mice that had undergone superovulation and were inseminated with preincubated spermatozoa and maintained under 5% CO_2_ at 37°C. The final concentration of spermatozoa in the insemination medium was ~150 spermatozoa/µL. Approximately 5‐6 hours after insemination, zygotes were transferred to KSOM for further cultivation.

### 2.6 Derivation of mouse PSCs from SCNT Blastocysts

Hatched blastocysts obtained from the In vitro fertilization (IVF) and SCNT groups both with and without RS‐1 treatment were placed on mitotic inactivated mouse embryonic fibroblast (MEF) feeder cells in mouse embryonic stem cell (mESC) culture medium to form outgrowths. DMEM/F12 containing 20% KSR, 0.1 mmol/L β‐mercaptoethanol, 1% non‐essential amino acids, 100 units/mL penicillin, 100 µg/mL streptomycin (all products from Gibco/Invitrogen) and 1.5 × 10^3^ units/mL recombinant mouse leukaemia inhibitory factor (Chemicon) was used as the mESC culture medium. Outgrowths were first mechanically transferred to new MEF feeder cells and then passaged using trypsin‐EDTA. All of the established mouse PSC lines were monitored and characterized by morphological examination and alkaline phosphatase staining. Alkaline phosphatase activity was assessed by histochemical staining. Colonies were fixed in 4% paraformaldehyde at room temperature for 1 minutes, washed twice with PBS and stained with an alkaline phosphatase substrate solution (10 mL of FRV‐alkaline solution, 10 mL of naphthol AS‐BI alkaline solution; alkaline phosphatase kit, Sigma‐Aldrich) for 30 minutes at room temperature. Alkaline phosphatase activity was detected colourimetrically by light microscopy.

### 2.7 Embryo transfer experiment

Somatic cell nuclear transfer embryos cultured to the 2‐cell stage under RS‐1 treatment or non‐treatment conditions were transferred into the oviduct of pseudopregnant female ICR mice that had been mated with a vasectomized male the night before transfer (0.5 days postcoitum [dpc]). Caesarean section was carried out at day 19.5 dpc and the surviving pups were fostered by lactating ICR females.

### 2.8 Array based comparative genomic hybridization

The ESCs and PSCs derived from experimental groups (IVF, SCNT and SCNT‐RS‐1) were washed in PBS prior to loading into a PCR tube containing lysis buffer. Genomic DNA was isolated by using a DNA multisample kit (Thermo Fisher Scientific) and stored at −80°C until being used in experiments. Whole‐genome amplification was performed using the GenomePlex Whole‐Genome Amplification (WGA) kit (Sigma‐Aldrich) to achieve the representative amplification of genomic DNA. Array based comparative genomic hybridization (aCGH) was performed using a SureScan unrestricted high‐definition CGH microarray (Agilent Technologies). After the scanning the microarray slides, the images were analysed using the Feature Extraction software provided by Agilent as both a standalone programme and an integral component of CytoGenomics software (Agilent).

### 2.9 Immunostaining

One‐cell and 2‐cell cloned embryos were washed in PBS containing 0.1% polyvinyl alcohol (PVA; Sigma‐Aldrich) and then fixed with 4% (w/v) paraformaldehyde at room temperature for 30 minutes. The embryos were next washed in PBS/PVA, permeabilized and blocked in PBS containing 0.1% Triton with 1% bovine serum albumin (BSA) overnight at 4°C. They were washed twice in PBS containing 0.1% BSA the following day, incubated with the primary antibody at room temperature for 2 hours in PBS‐0.1% BSA, washed twice at room temperature in 0.1% PBS‐0.1% BSA, incubated with the secondary conjugated antibody in PBS‐0.1% BSA at room temperature for 1 hour, washed as indicated above, stained with DAPI for 30 minutes and used for confocal microscopy. Staining and analysis was performed using antibodies recognizing phospho‐γH2AX (05‐636, clone JBW 301, Millipore, 1:1,000), RAD51 (PC130, EMD Millipore, 1:1,000), H3K9me3 (ab8898, Abcam, 1:1,000), and Oct4 antibody (sc5279, Santa Cruz, 1:200), which were diluted in PBS‐0.1% BSA buffer. The same conditions were maintained between different samples.

### 2.10 Comet assay

DNA damage in the 1‐cell embryos generated from IVF or SCNT was assessed at different time (5 and 8 hours after insemination or SCNT) by DNA comet length using a neutral version of the comet assay,^37^, ^38^ as detailed in the manufacturer’s instructions of the CometAssay^®^Kit (Trevigen, Inc).

To maximize cell lysis efficiency, the zona pellucida of the embryos was removed. After the embryos were suspended in 1% low‐melting‐point agarose (LMA) at 37°C, the LMA‐embryo mixture was pipetted onto slide provided with the CometAssay^®^ Kit. The slides were incubated at 4°C in the dark to achieve complete adherence and then immersed in chilled Trevigen cell lysis solution overnight. The next day, the slides were removed from the lysis solution and gently immersed in 4°C 1X Tris‐acetate‐EDTA (TAE) buffer for 30 minutes to wash out residual lysis solution. Next, the slides were subjected to electrophoresis for 40 minutes at 30 V. The slides were then immersed in 1 M ammonium acetate solution at RT for 25 minutes, followed by immersion in 75% ethanol for fixation at RT for 25 minutes. After the fixation step, the slides were incubated at 40°C for at least 40 minutes until the LMA was fully dried and then stained with 1X SYBR^®^ Green I Staining Solution for 5 minutes in the dark. The stained slides were observed under a Nikon ECLIPSE TE2000‐V fluorescence microscope. To quantify individual embryo comets, we scored each comet according to tail length.

### 2.11 Quantitative reverse transcription‐polymerase chain reaction (RT‐PCR)

Total RNA of each group was extracted from twenty 1‐, 2‐ or 4‐cell stage embryos using a TRIzol reagent (Invitrogen). cDNA was synthesized from total extracted RNA using the LeGene Premium Express 1st strand cDNA Synthesis System (LeGene Biosciences) according to the manufacturer's instructions (to final 20 μL of volume). Quantitative real‐time PCR was performed with 2 μL cDNA solution (from 2 embryos) by using TOPrealTM qPCR 2X PreMIX (Enzynomics) on the Bio‐Rad CFX96™ Real‐time PCR machine (Bio‐Rad). The ΔΔ*C*_t_ method was applied to normalize expression levels of each gene to those of *Gapdh*. The following primers were used for Quantitative real‐time PCR: mouse *Rad51* forward 5′‐ CAG TGG AGG CTG TTG CTT AT‐3′, mouse *Rad51* reverse 5′‐CAG CTC TTT GGA GCC AGT AG‐3′, mouse *Gapdh* forward 5′‐ AAT GGT GAA GGT CGG TGT G‐3′, mouse *Gapdh* reverse 5′‐ ACA AGC TTC CCA TTC TCG G ‐3′.

### 2.12 Library preparation and single‐cell‐RNA sequencing

For the RNA‐Seq analysis, the DriveMap^TM^ library preparation method (Selecta) was used. For cDNA synthesis, each embryo was lysed in 1× TCL lysis buffer, deposited in separate wells of a TurboCapture 96 mRNA plate (Qiagen) and incubated at room temperature for 1 hour After washing the plate three times with cold TCW washing buffer (Qiagen), 10 µL of RT reaction master mix solution was added. The plate was then subjected to incubation at 50°C for 40 minutes, followed by RT inactivation at 95°C for 5 minutes.

For the first round of gene‐specific primer (GSP) extension, 10 µL of cDNA and 10 µL of multiplex DNA polymerase master reaction mix (pool of forward GSP) were mixed and incubated for 30 minutes at 64°C. After the primer removal reaction, the plate was incubated for 30 minutes at 37°C and 5 minutes at 95°C. The second extension was conducted under the same conditions as the first extension, and the reaction was terminated by the addition of primer removal reagent mix and incubation at 37°C for 30 minutes. Using a unique combination of Fwd and Rev IND PCR primers, the first (20 cycles) and 2nd (9 cycles) rounds of PCR were performed to amplify the DNA. The amplified DNA was purified and subjected to sequencing using the NextSeq500 Illumina platform.

### 2.13 Analysis of RNA‐seq data

RNA‐Seq data were analysed by using either the ROSALIND tool (OnRamp) or public resources. The STAR tool (v2.5.2b, https://github.com/alexdobin/STAR) was used to map raw paired‐end reads to the mouse mm9 genome assembly. Using the STAR outcome files, Cuffnorm of Cufflinks (v2.2.1) was used to determine the FPKM values of genes in the SCNT, SCNT‐RS and IVF samples.^39^ Genes were considered differentially expressed at a fold change >3 and FPKM >3. The clustering and visualization of DEGs were carried out with the gplots package (v3.0.1.1) in R (v3.3.2) (https://www.R‐project.org/). Gene ontology (GO) (biological process) analyses of the DEGs were performed using the DAVID tool (v6.8).^40^ Using the ClueGO tool (v2.5.1) ^41^ plug‐in of Cytoscape (v3.6.1),^42^ KEGG pathway analyses of the DEG clusters in heatmaps were performed. *P*‐values were calculated with right‐sided hypergeometric tests. Benjamin‐Hochberg adjustment was used for the correction of multiple tests. KEGG pathways with *P*‐values <.05 were considered statistically significant.

### 2.14 Statistical analysis and reproducibility

All data presented with error bars came from consist of at least three independent SCNT experiments (RNA‐seq analysis is exceptional and not repeated). Representative embryos were imaged for all strains, and images of the same embryo were displayed in the figures. The results are presented as the mean ± SEM. GraphPad Prism 5.0 software was used to calculate statistical significance with Student’s *t* test for relevant figures, as specified in the figure legends. Embryonic development was analysed by one‐way ANOVA with Duncan’s test using SAS software, while implantation and ESC‐derivation rates were analysed with the chi‐square test. *P* < .05 was regarded as statistically significant.

## 3 RESULTS

### 3.1 RS‐1 recovered the developmental insufficiency of SCNT‐derived mouse embryos

To confirm the insufficiency of embryonic development from SCNT‐derived embryos, the rates of blastocyst formation and RAD51 activity (DDR ) were compared in SCNT‐ and IVF‐derived embryos. As shown in Figure S1, unlike IVF embryos, the majority of SCNT embryos failed to develop into blastocysts and degenerated (93.0 ± 1.4% vs 33.7 ± 0.4%, *P* < .05). To determine the reason for this difference, we analysed the expression of *Rad51,* a well‐known regulatory factor for the DDR and genomic instability. Interestingly, the relative mRNA expression of *Rad51* in SCNT‐derived 1‐cell embryos was lower than that in IVF‐derived embryos and was not recovered in SCNT‐derived 4‐cell embryos. We hypothesized reduced RAD51 activity during SCNT may have a negative role on further embryonic development. Therefore, to analyse whether increased RAD51 activity overcame developmental insufficiency and reduced genomic stability, 0‐15 µmol/L RS‐1 was added to SCNT embryos for 22 hours after SCNT and SrCl_2_ activation. Treatment with RS‐1 reduced the developmental block of SCNT‐derived 2‐cell embryos and subsequently increased embryonic development up to the blastocyst stage. In particular, embryonic morphology and development in the 10 µmol/L RS‐1‐treated group were much improved over those in the other groups (Figure 1A,B). However, there was no significant difference in the total cell number and inner cell mass number between blastocysts in the SCNT and SCNT plus RS‐1‐treated (SCNT‐RS‐1) groups (Figure S2).

Based on a previous report,^32^ G1‐phase, S‐phase and G2‐phase 1‐cell embryos were harvested at 5, 8 and 12 hours post‐activation, respectively. To determine the differences in chromatin structure during reprogramming, the numbers of γH2AX spots (a marker of DSB) and RAD51 spots were analysed in IVF‐derived and SCNT‐derived 1‐cell embryos (at G2‐phase). Interestingly, the number of γH2AX spots in SCNT embryos was much lower than that in IVF embryos, and the RS‐1 treatment of SCNT embryos (SCNT‐RS‐1 group) increased γH2AX spots compared to non‐treated SCNT embryos (SCNT group) (Figure 1C). In most cases, the number of RAD51 spots showed similar patterns to those of γH2AX and Rad51 spots overlapped with γH2AX spots. In addition, the number of SCNT embryos with RAD51 spots was lower than that of IVF embryos, and this number was greatly increased in the SCNT‐RS‐1 group (Figure 1D).

To compare the genomic DNA instability of the IVF, SCNT and SCNT‐RS‐1 group during reprogramming, the frequency of DNA fragmentation was analysed by applying the comet assay. The lengths of the DNA fragments (comet tail lengths) observed at G1‐phase in the 1‐cell embryo were very similar among the IVF, SCNT and SCNT‐RS‐1 groups. However, the comet tail length at S‐phase was clearly distinguishable between IVF embryos and SCNT embryos. SCNT embryos at S‐phase clearly showed notably longer comet tails than IVF embryos, indicating that the genomic DNA of SCNT embryos was severely damaged in S‐phase of the 1‐cell stage. In contrast, the comet tail lengths of the SCNT‐RS‐1 group were similar or even shorter than those of the IVF samples (Figure 1E,F, Figure S3). These observations suggest that RS‐1 may have resolved the DNA damage stress caused in SCNT embryos by progression through S‐phase of the 1‐cell stage.

### 3.2 Improved embryonic development of SCNT‐derived mouse eggs may be related to changes in gene expression induced by RS‐1 treatment

**FIGURE 2:**
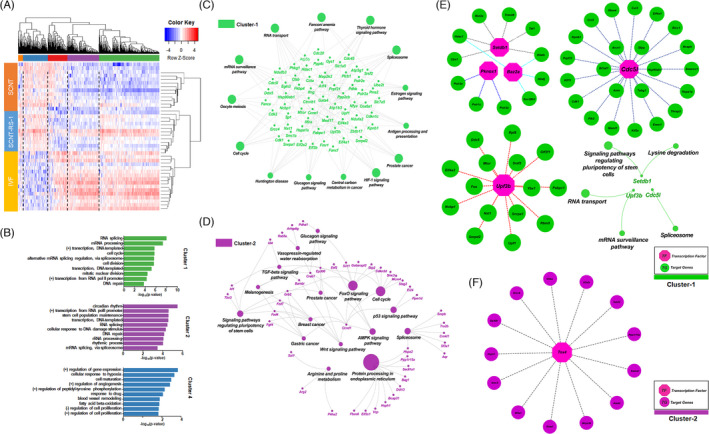
Single‐cell RNA‐Seq analysis of SCNT, SCNT‐RS‐1 and IVF 2‐cell embryos. A, Heatmap showing the unsupervised hierarchical clustering of differentially expressed genes (DEGs, FPKM >3, fold change >3) for each sample. Colours represent the *z*‐scores of gene expression on a log2 scale. B, Gene Ontology analysis of the gene clusters in the heatmap. The 10 biological process Gene Ontology terms with the lowest *P*‐values are shown. Green bars, purple bars and blue bars represent cluster 1, cluster 2 and cluster 4, respectively. C, D. KEGG pathway analysis of the genes of cluster 1 and cluster 2. Green: cluster1, Purple: cluster2. E, F. Transcription factors/epigenetic regulators identified in clusters 1 (pink and green colour) and 2 (pink and purple colour) and their target genes identified as DEGs. The KEGG pathway analysis of Setdb1, Upf3b and Cdc5l is illustrated

Next, we employed a single‐cell RNA‐Seq approach to examine the changes in gene expression induced by RS‐1 treatment. To that end, at least 10 2‐cell embryos from the SCNT, SCNT‐RS‐1 or IVF groups were individually subjected to RNA‐Seq analysis. Our analyses revealed that RS‐1 treatment changed the gross gene expression pattern in SCNT‐2 cells to a pattern close to that in IVF 2 cells (Figure 2A). In a non‐supervised hierarchical analysis, five major gene clusters were identified and subjected to GO analysis. As shown in Figure 2B, clusters 1 and 2, containing genes showing an expression profile similar to that of IVF 2 cells, exhibited GO terms related to RNA processing, transcription, the cell cycle and DNA repair. On the other hand, cluster 4, comprising genes showing an expression profile close to that of SCNT‐2 cells, included GO terms related to transcription, cell maturation and proliferation. Together, these results suggest that RS‐1 exposure somehow activates the gene expression programme (at both transcriptional and translational levels) required for early embryonic development. However, as indicated in cluster 4, RS‐1 by itself is insufficient to activate entire transcriptional programme for early embryonic development. Next, to examine biological functions altered by RS‐1, KEGG pathway analysis was performed. As shown in Figure 2C, the spliceosome, RNA transport and mRNA surveillance pathways were markedly changed by RS‐1. Oocyte meiosis‐ and cell cycle‐related genes were also restored by RS‐1. The spliceosome and cell cycle pathways were included in the genes of cluster 2 as well (Figure 2D). Finally, given that epigenetic regulators (ERs)/TFs regulate ZGA,^43^ we further investigated differentially expressed ERs/TFs and their downstream target genes in clusters 1 and 2. The analysis revealed that several ERs/TFs including Setdb1, Pknox1, Baz2a, Cdc5l, Upf3b and Tox4 were restored in the SCNT‐RS‐1 embryos (Figure 2E,F). The H3K9 methyltransferase Setdb1 was identified as a regulator of pluripotency in the KEGG pathway analysis. Consistent with our analysis, previous studies have shown that a maternal origin of Setdb1 in oocytes ensures normal meiosis, preimplantation development and epigenetic reprogramming.^44^, ^45^

### 3.3 RS‐1 improves the derivation of ESCs and the production of cloned offspring

**FIGURE 3:**
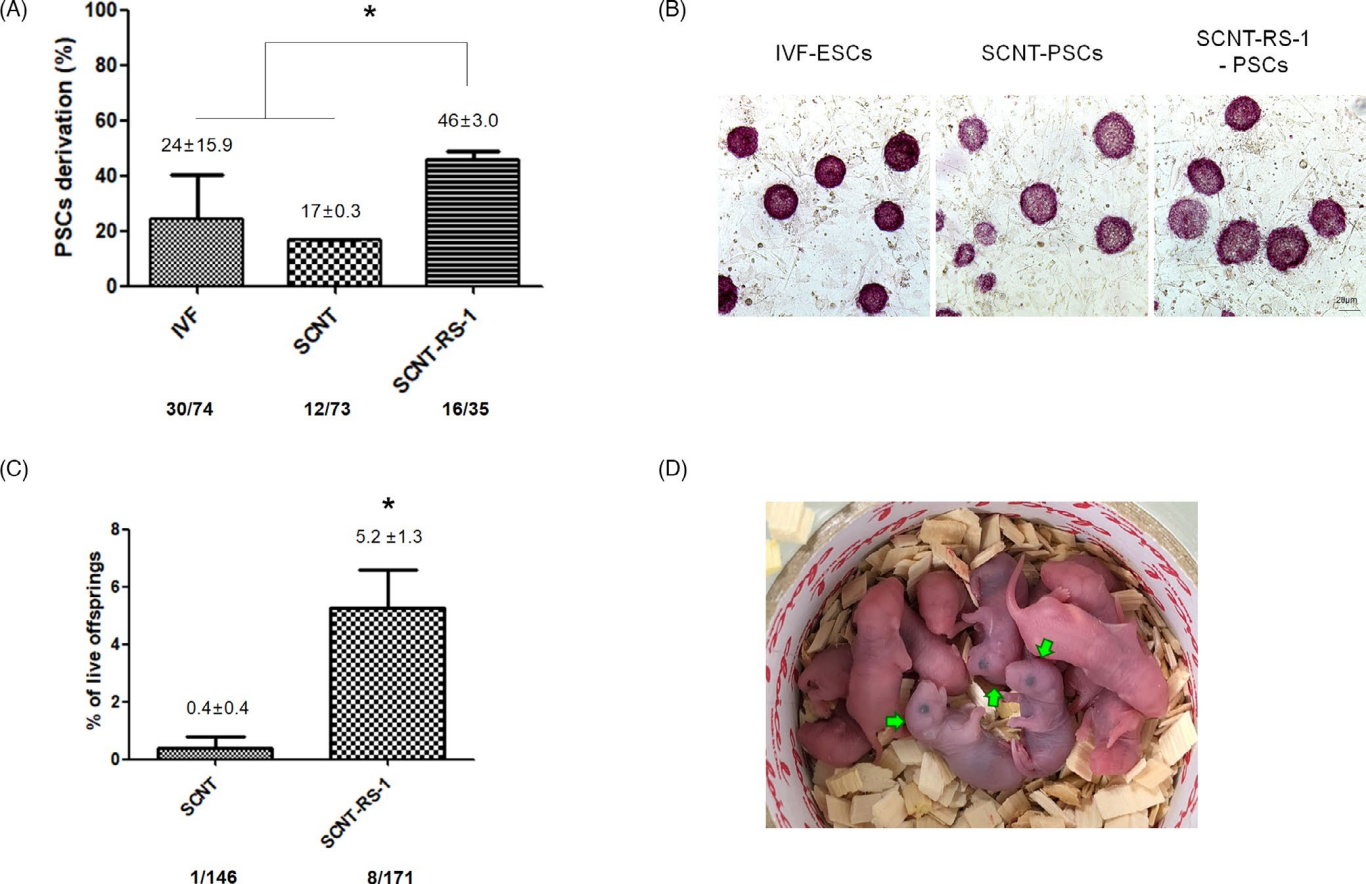
SCNT‐PSCs and healthy pups derived from the RS‐1 treatment of cloned embryos. A, Efficiency of IVF‐ESC, SCNT‐PSC, and SCNT‐RS‐1‐PSC derivation. The efficiency of SCNT‐PSC derivation was analysed based on the total number of blastocysts cultures on mitotically inactivated MEF feeder cells. ESC and PSC derivation was performed three times (^*^ *P* < .05). B, Photograph of ESCs and PSCs from the IVF, SCNT, and SCNT‐RS‐1 groups. Alkaline phosphatase staining (AP staining) represents colonies. C, The efficiency of SCNT and SCNT‐RS‐1 cloned pup derivation was analysed based on the total number of embryos transferred to the pseudopregnant mice (^*^ *P* < .05). D, Normal growth was observed during nursing in the cloned pups (green arrow). Cloned pup derivation was performed five times

RS‐1 plays a positive role in the embryonic development of SCNT‐derived embryos up to the blastocyst stage; therefore, we analysed the effect on postimplantation development. First, as shown in Figure 3A,B, a higher derivation rate of mESCs was obtained in the SCNT‐RS‐1 group than in the RS‐1 non‐treated (SCNT) and IVF groups (46 ± 3.0% vs 17 ± 0.3% and 24 ± 15.9%, respectively, *P* < .05). In addition, treatment of RS‐1 improved the derivation efficiency on the IVF embryos and it may also have a beneficial effect on the development and ESC‐derivation on IVF embryos (44 ± 6.0% vs 28 ± 5.8%, *P* <.05) (Figure S4).

To analyse the long‐term safety of RS‐1 treatment during embryonic development, we performed array comparative genomic hybridization (CGH) to identify genetic variations in ESCs derived from the IVF, SCNT and SCNT‐RS‐1 groups. As shown in Figure S4, in ESCs from both the SCNT and SCNT‐RS‐1 groups, the gain and loss of copy number variation (CNV) during reprogramming and prolonged periods in culture were not different compared to those in the IVF group (Figure S5). In particular, these data may show that there was no harmful effect of RS‐1 treatment on genetic stability because there was no difference between the RS‐1‐treated and non‐treated groups. In addition, to compare the embryonic quality of cloned embryos according to RS‐1 treatment, we analysed the delivery rate after embryo transfer. The number of offspring in the SCNT‐RS‐1 group was significantly increased compared to that in the SCNT group (5.2 ± 1.3% vs 0.4 ± 0.4%, *P* < .05; Figure 3C,D). All pups were normal morphology, healthy and well‐grown into adult.

### 3.4 RS‐1 overcomes the developmental block of mouse SCNT eggs through a mechanism independent of epigenetic regulators

According to the above results, RS‐1‐treated SCNT‐2‐cell embryos showed a higher rate of embryonic development beyond the 2‐cell stage. In fact, developmental arrest at the 2‐cell stage (2‐cell block) is the main obstacle that must be overcome for successful SCNT reprogramming. Previous reports have indicated that the abnormal regulation of histone lysine methylation during SCNT may be a resistant reprogramming barrier, and the injection of *histone lysine demethyltransferase (Kdm 4a)* mRNA into reconstituted eggs after SCNT can overcome the 2‐cell block and contribute to successful embryonic development. To analyse the mode of action of RS‐1 treatment and its synergic effect with injection of *Kdm 4a* mRNA, we designed a series of analyses such as the comparison of RNA‐seq results between RS‐1‐treated and *Kdm 4a* mRNA‐injected groups as well as the comparison of embryonic development between RS‐1‐treated groups with or without *Kdm 4a* mRNA treatment.

**FIGURE 4:**
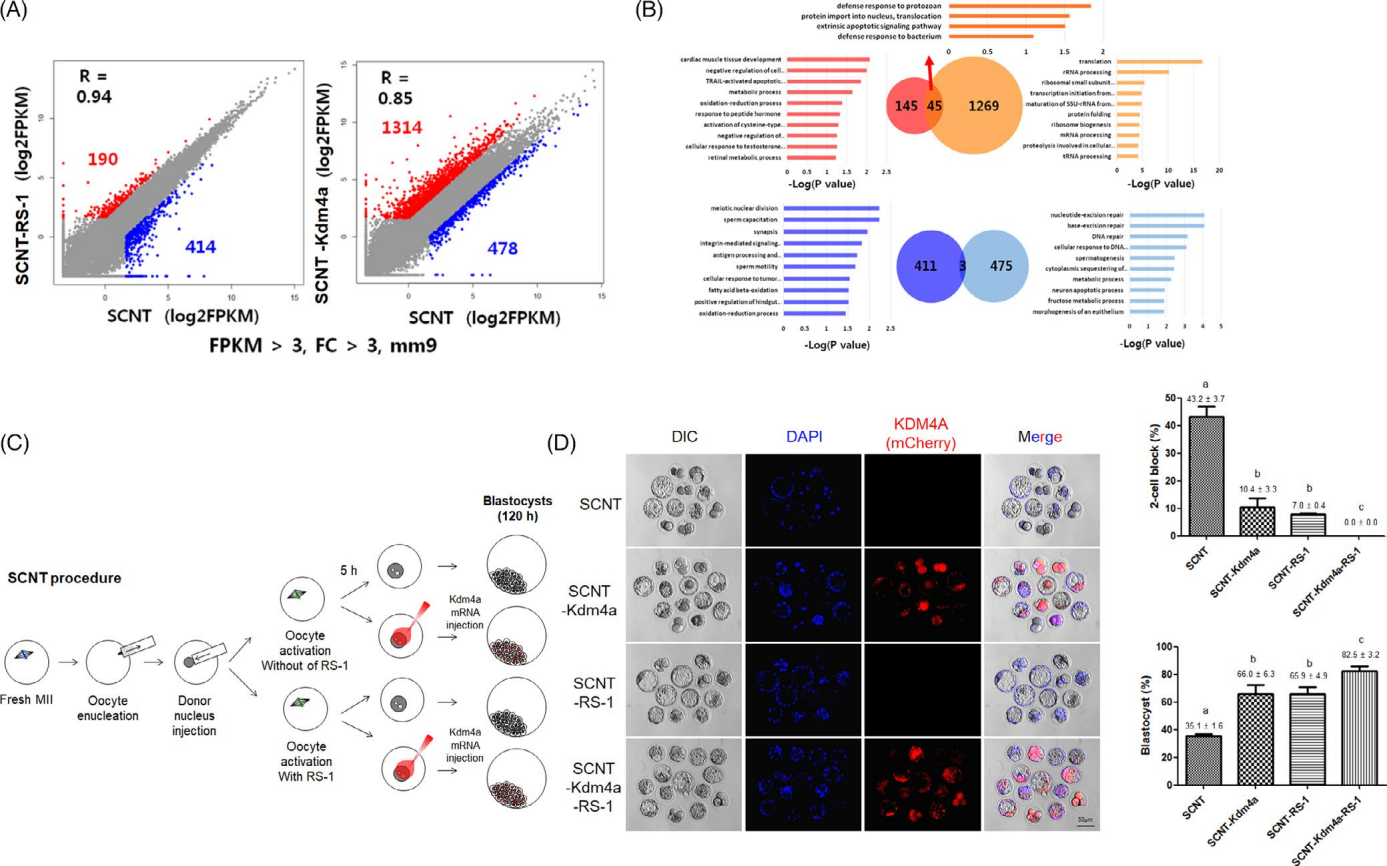
Comparison of RNA‐seq results and embryonic development between RS‐1‐treated and *Kdm 4a* mRNA‐injected groups. A, B, Scatter plots representing the DEGs identified in SCNT‐RS‐1 vs SCNT and SCNT‐Kdma4a vs SCNT. The red and blue dots in the plot represent upregulated and downregulated DEGs in the comparison, respectively. Gene ontology analysis of the upregulated or downregulated DEGs identified in each comparison; 45 and 3 genes were commonly upregulated and downregulated, respectively, in both comparisons. The cut‐off values are set at FC >3 and FPKM>3. C, Illustration of the experimental design for examining the synergistic effect of RS‐1 treatment after the injection of *Kdm4a* mRNA after SCNT. D, Synergistic effect on the efficiency of blastocyst formation following RS‐1 treatment and/or *Kdm4a* mRNA injection after SCNT. The different superscripts on the bars indicate significantly different value (*P* < .05). This experiment was repeated more than 3 times and the number of embryos was more than 20 (per each group)

First, we performed another RNA sequencing analysis using 2‐cell embryos (50 embryos/sample) from the SCNT and SCNT‐RS‐1 groups, and the data were analysed together with previous RNA‐seq results from the SCNT and SCNT‐Kdm4a injected (SCNT‐Kdm4a) groups.^27^ As shown in the Figure 4A,B, compared to the SCNT group, 190 genes were upregulated and 414 genes were downregulated in the SCNT‐RS‐1 group. In addition, 1314 genes were upregulated and 478 genes are downregulated the in SCNT‐Kdm4a group. However, only 45 upregulated (3.08%, 45/1459) and 3 downregulated genes (0.34%, 3/889) were common to the two groups. Notably, *Acap3, Faiml, Gipc1, Lmx1a* and *TnFRSf12a*, which are involved in cell survival and tissue regeneration, were upregulated in 2‐cell embryos from both the SCNT‐Kdm4a and SCNT‐RS‐1 groups. In addition, *chit1, Ifng* and *Lat2*, which are involved in the immune response and *Bcl3, Bop1, Fanca, Gpr161, P2ry1, Slc22a20, Sva, Yif1b* and *Zc3h12a*, which are involved in the maintenance of pluripotency, the cell cycle, DNA repair, tumour suppression, mitochondrial metabolism, germ cell proliferation, calcium oscillation, the organization of the Golgi architecture, and maintenance of endothelial homeostasis, were also upregulated. In contrast, the expression of the spermatogenesis‐related gene *Xlr5c* was downregulated in 2‐cell embryos from both the SCNT‐Kdm4a and SCNT‐RS‐1 groups (Table S1). In addition, in another round of experiments, the rate of 2‐cell block in the SCNT‐RS‐1 and SCNT‐Kdm4a groups were significantly lower than that in the SCNT group (7.8 ± 0.4% and 10.4 ± 3.3% vs 43.2 ± 3.7%, *P* < .05). None of the reconstituted eggs showed a 2‐cell block in the SCNT‐RS‐1+Kdm 4a group (*P* < .01). Additionally, embryonic development up to the blastocyst stage was greatly increased in the SCNT‐RS‐1‐Kdm 4a group (Figure 4C,D). This experiment was repeated three time (≥20 SCNT embryos/each group/trial). These results may suggest that the two treatments have different modes of action and exert synergistic effects to overcome the developmental block of mouse SCNT eggs.

## 4 DISCUSSION

Somatic cell nuclear transfer (the oocyte‐mediated reprogramming of somatic cells) is an amazing tool developed in recent decades to test the role of epigenetic regulation in developmental programming and to generate genetically matched PSCs by reprogramming.^3^, ^24^ One of the major obstacles to successful SCNT technologies is developmental arrest due to abnormal gene expression at the stage of embryonic genome activation, which we partly overcame by epigenetic modification in our previous study.^27^ In the present study, we found that low activity of RAD51 during genomic reprogramming after SCNT causes a decrease in DNA repair by homologous recombination and an increase in genetic instability, ultimately resulting in developmental arrest. In fact, supplementation with RS‐1 during reprogramming recovered RAD51 activity and gene expression in reconstituted eggs and further improved embryonic development and the derivation efficiency of PSCs.

Homologous recombination is the most widely used mechanism for the error‐free DNA repair of double‐strands breaks in somatic and germ cells in mammals. In particular, RAD51 plays a major role in the homologous recombination of DNA during DSB repair and regulates normal DNA replication.^46^ In our preliminary study, SCNT‐derived eggs showed poor embryonic development compared to IVF‐derived eggs. Interestingly, these embryos also exhibited lower expression of *Rad 51* at the pronuclear (1‐cell stage) and 4‐cell stages (Figure S1). To evaluate the effect of DNA repair by RAD51 on reprogramming, embryonic development and genomic modification, RS‐1 (an activator of *Rad51*)‐treated SCNT eggs were analysed. Treatment with RS‐1 overcame the developmental block at the 2‐cell stage, and embryonic quality and development were greatly increased under treatment at 10 µmol/L concentration compared with the other treatments. In further analysis using 10 µmol/L concentration of RS‐1, the foci of RAD51 were increased in the treated SCNT eggs and γH2AX spots were also increased (Figure 1A‐D). The γH2AX foci that form at sites of DNA damage serve to recruit repair proteins and it has been suggested that this leads to recombination and conformational changes in chromatin.^47^, ^48^, ^49^ These results may suggest that RS‐1 upregulates RAD51 activity, which greatly increases the genomic modification of nuclear donors by inducing DSBs and HDRs when exposed to oocyte cytoplasm. Similarly, IVF embryos exhibit a greater number of DSBs by the 1‐cell stage than parthenogenetic and SCNT embryos.^50^ Additionally, high HR‐related gene expression in iPSCs has been achieved through reprogramming.^51^, ^52^ Therefore, these results may suggest that successful reprogramming during fertilization and the dedifferentiation of somatic cells in oocyte cytoplasm require a proper DSBs in the genome and its repair system.

It is well established that reprogrammed human PSCs contain a number of protein‐coding mutations, which may be preexisting mutation or arise during reprogramming process.^53^, ^54^ To test the safety of RS‐1 treatment on SCNT reprogramming, we analysed chromosome instability using the comet assay in SCNT eggs and array CGH in SCNT‐PSCs and then compared the results with those for IVF eggs and IVF‐ESCs. In 1‐cell eggs, the comet tail lengths of the three groups (IVF, SCNT and SCNT‐RS‐1) were not different in G1‐phase, but they were greatly increased in the S‐phase of SCNT eggs compared to those of IVF and SCNT‐RS‐1 eggs (Figure 1E). These data may suggest that a large amount of genetic instability exists due to DNA damage that occur during SCNT reprogramming, which is a reason for the developmental arrest observed in SCNT eggs. DNA damage can be greatly reduced by treatment with RS‐1 (Figure 1E and Figure S2). However, we did not find much differences in CNVs in ESCs and PSCs among all three groups (Figure S3). This result is very similar to previous findings indicating that CNVs and InDels in human SCNT‐PSCs and IVF‐ESCs show no statistically significant differences.^55^ In addition, it may prove that the increase in RAD51 activity induced by RS‐1 treatment during SCNT reprogramming has no effect on genetic stability after embryonic development and stem cell derivation.

RS‐1‐treated SCNT eggs have been shown to overcome developmental arrest and present good embryonic development, similar to that of IVF‐derived embryos. Following RNA‐seq analysis using a single embryo, RS‐1‐treated SCNT‐2‐cell embryos showed the reactivation of a large number of genes that were not expressed in SCNT‐2‐cell embryos, and their expression patterns were also much more similar to those of IVF embryos (Figure 2A). KEGG analysis suggested that upregulation of RNA and protein processing‐, cell cycle‐ and epigenetic regulation‐related genes in clusters 3 and 4 (Figure 2B). In addition, RS‐1‐treated SCNT embryos showed a higher derivation rate of PSCs than non‐treated SCNT embryos in vitro as well as greater production of viable offspring in vivo (Figure 3). Therefore, we suggest that treatment with RS‐1 during SCNT reprogramming may upregulate the expression of previously repressed genes by recovering RAD51 activity for HDR‐related DNA repair.

In our previous report, we found insufficient gene expression (referred to as reprograming‐resistant regions, RRRs) due to the presence of epigenetic barriers such as histone H3 lysine 9 trimethylation (H3K9me3) in SCNT embryos, and we found that the reduction of barrier activity through the ectopic expression of the histone demethylase KDM4A could fully or partially activate some genes of RRRs and greatly improve SCNT embryo development.^27^ In the present study, we performed comparative studies of embryos derived from two systems, RS‐1‐mediated and Kdm4a‐mediated SCNT, to understand their mode of action. In the first study, the gene expression of 2‐cell embryos from the SCNT‐RS‐1 and non‐treated SCNT control groups was analysed by RNA sequencing, and the results were compared with those from SCNT‐Kdm4a‐injected (SCNT‐Kdm4a) and non‐injected SCNT control groups.^27^ Interestingly, compared with the same control (SCNT‐only group), 190 and 1314 genes were upregulated in the SCNT‐RS‐1 and SCNT‐Kdm4a groups, respectively. Additionally, 414 and 478 genes were downregulated in the SCNT‐RS‐1 and SCNT‐Kdm4a groups, respectively. However, only 45 genes out of 1504 upregulated genes and 3 genes out of 889 downregulated genes were commonly expressed in both systems. Additionally, the functional groups of differentially expressed genes were very different between the SCNT‐RS‐1 and SCNT‐Kdm4a groups (Figure 4A,B). In another direct comparison study, the developmental block at the 2‐cell stage (embryonic gene activation, EGA) was shown to be overcome to a large extent, and blastocyst formation was improved in the SCNT‐Kdm4a and SCNT‐RS‐1 groups. More interestingly, when the two systems were coapplied in mouse SCNT reprogramming (SCNT‐ Kdm4a + RS‐1 group), we found a synergistic positive effect on embryonic development. On the basis of these results, we suggest that the two systems (RS‐1 treatment and Kdm4a mRNA injection) may exhibit different modes of action in the reactivation of gene expression and play a positive role in SCNT reprogramming when applied either separately or in combination. Additionally, it is important to find an effective small molecule that can overcome the reprogramming barrier because technical difficulties are a critical obstacle to the use of SCNT‐PSCs as a major source for cell therapy.

In the present study, we observed that transcriptional activity has been altered during SCNT reprogramming and it also may be caused by a lower activity of DNA repair system, resulted in developmental block on further embryonic development. And, we determined the effect of upregulation of RAD51 by treatment with RS‐1 on the role of DSB repair during SCNT reprogramming and the improvement of embryonic development by recovery of gene expression. Therefore, we suggest that RS‐1 treatment would be an efficient, safe and simple protocol for improving the efficacy of SCNT technology.

## CONFLICT OF INTEREST

The authors indicate no potential conﬂicts of interest.

## AUTHOR CONTRIBUTIONS

DRL and KSP conceived the project. ARL, SHS, KSP and DRL designed the experiments. ARL performed mouse SCNT experiments. ARL, JHP, KHH, and HL conducted experiment data analyses. ARL, KSP and DRL wrote the manuscript.

## Supporting information

Supplementary Material

## Data Availability

All data that support the findings of this study are available from the corresponding author upon reasonable request. However, RNA‐seq data are available in GEO database at [https://www.ncbi.nlm.nih.gov/geo/query/acc.cgi?acc=GxxxxSE151074]. The GEO accession number: GSE151074.
