# Supplementary material for: Genome stabilization by RAD51‐stimulatory compound 1 enhances efficiency of somatic cell nuclear transfer‐mediated reprogramming and full‐term development of cloned mouse embryos
Source: Cell Prolif. 2021 May 21;54(7):e13059. doi: 10.1111/cpr.13059 (PMC8249786; doi:10.1111/cpr.13059)
Supplement: Supplementary file 1 — Supplementary Material [file CPR-54-e13059-s001.docx]

Lee et al. Supporting information

**Genome stabilization by RAD51 stimulatory compound-1 enhances efficiency of somatic cell nuclear transfer-mediated reprogramming and full-term development of cloned mouse embryos**

Ah Reum Lee^1,2^, Ji-Hoon Park^1^, Sung Han Shim^1^, Kwonho Hong^3^, Hyeonwoo La^3^, Kyung-Soon Park^1 #^, and Dong Ryul Lee^1,2 #^

^1^ Department of Biomedical Science, CHA University, Seongnam, Gyunggi-do 13488, Korea

^2^ CHA Advanced Research Institute, CHA University, Seongnam, Gyunggi-do 13488, Korea

^3^ Department of Stem Cell and Regenerative Biology, Konkuk University, Gwangjin-gu, Seoul 05029, Korea

^#^ To whom correspondence should be addressed: e-mail: [drleedr@cha.ac.kr](mailto:drleedr@cha.ac.kr) and [kspark@cha.ac.kr](mailto:kspark@cha.ac.kr)

Running Head: Efficient production of SCNT-embryos

Keywords: somatic cell nuclear transfer; homologous recombination repair; *Rad51*; RS-1; reprogramming


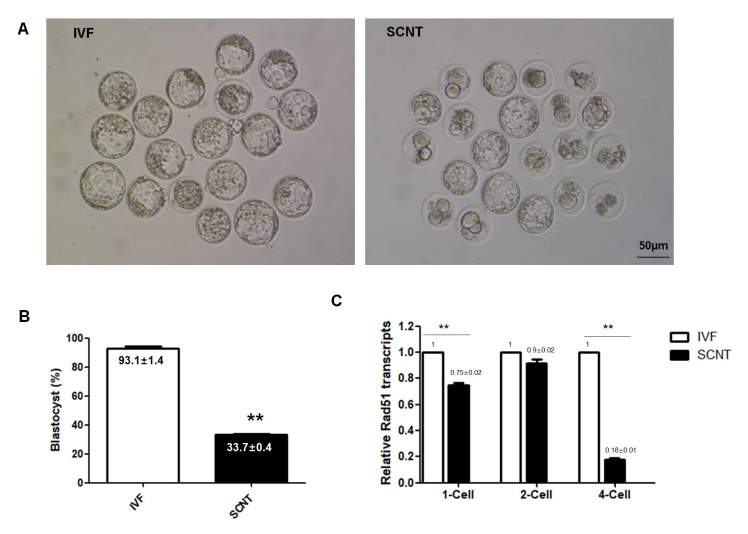


**Supplemental Figure 1. A and B.** Blastocyst formation in IVF and SCNT embryos. The data shown are from three independent experiments. The results indicated with bars are presented as the means±SEM. C, Quantitative real-time PCR comparing RAD51 mRNA levels in the IVF and SCNT groups at the 1-cell, 2-cell, and 4-cell stages. ^**^ on the bars indicates significantly different values (*p*<0.01).


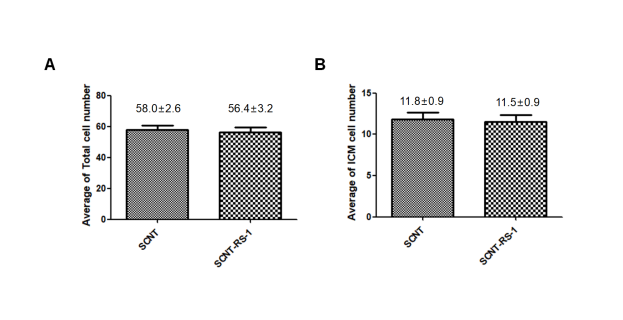


**Supplemental Figure 2** Total cell number and inner cell mass (ICM) counts and average numbers of embryos derived from the SCNT and SCNT-RS-1 groups. The number on the bar indicates the mean±SEM.


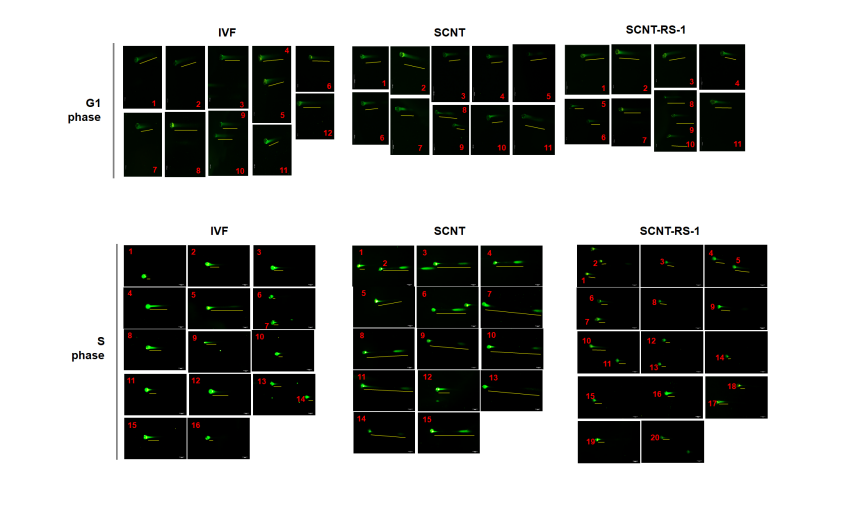


**Supplemental Figure 3.** All results of the neutral comet assay in G1-phase and S-phase in the IVF, SCNT, and SCNT-RS-1 groups. Comet images of 1-cell embryos stained with SYBR and observed under fluorescence microscope.

**IVF**

**IVF-RS-1**


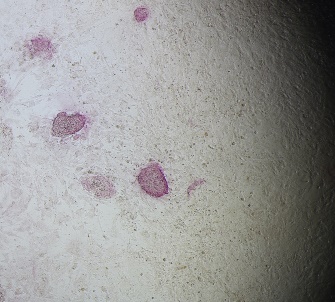

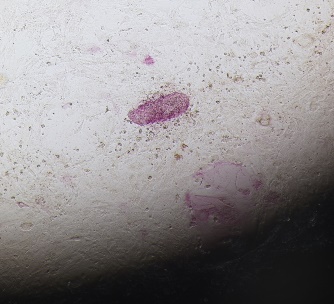

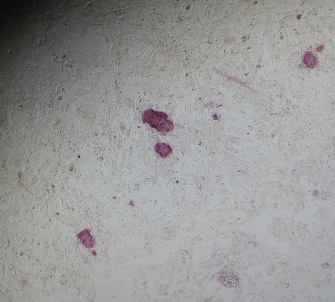

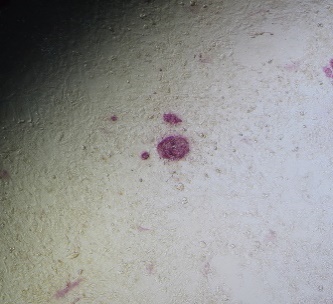

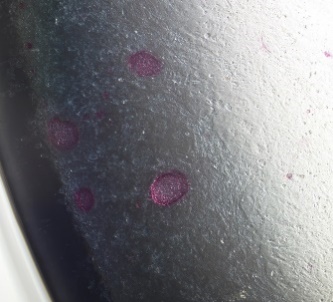

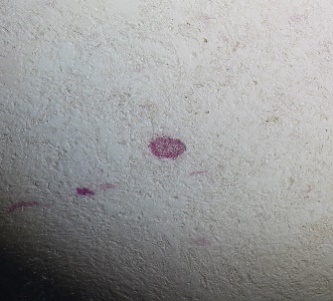

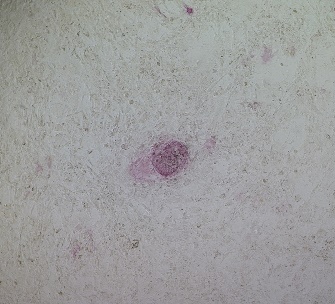

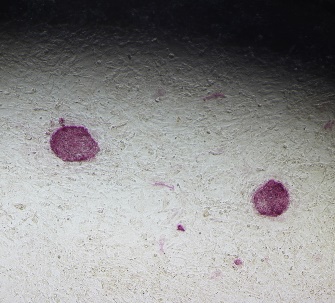

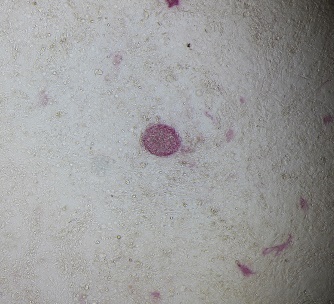

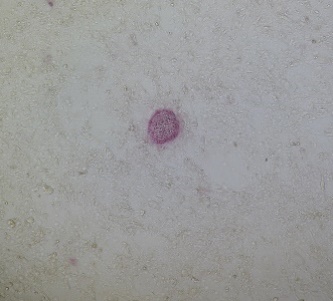

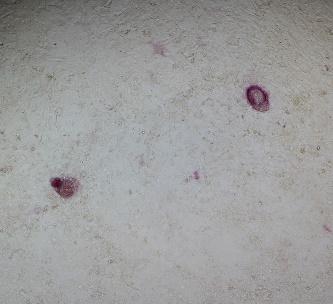

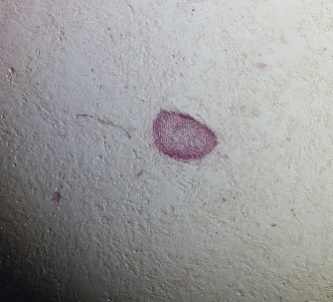

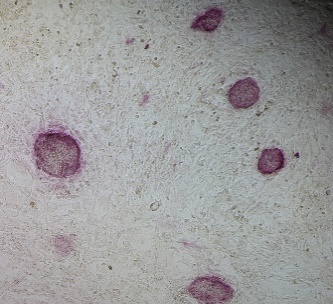

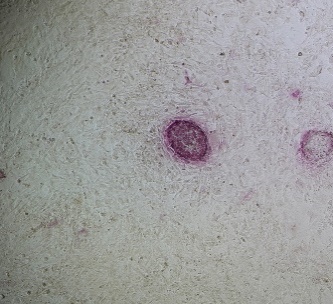

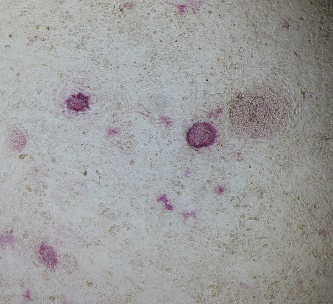

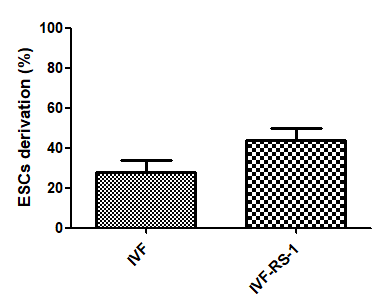


**28 ±5.8**

**44 ±6.0**

*****

**28/100**

**44/100**

**Supplemental Figure 4.** Efficiency of IVF-ESC and IVF-RS-1-ESC derivation. The efficiency of IVF-ESC derivation was analyzed based on the total number of blastocysts cultures on mitotically inactivated MEF feeder cells. ESC derivation was performed more than three times (^*^ *p*<0.05).


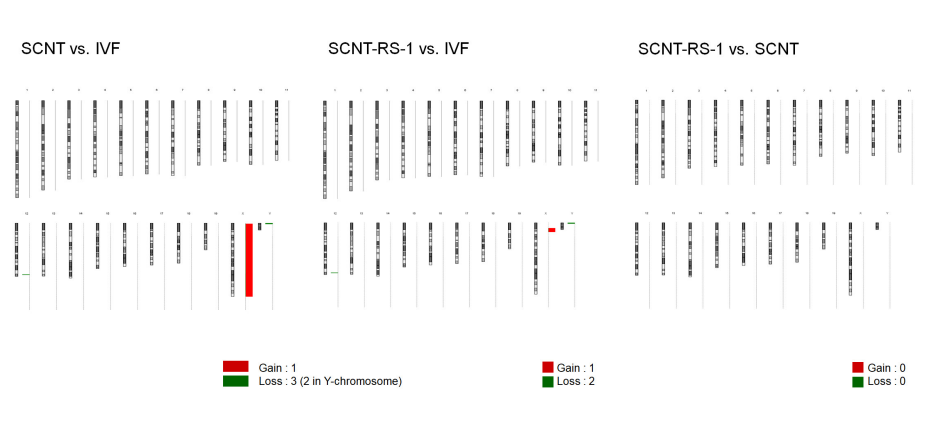


**Supplemental Figure 5.** Array CGH analysis of the entire chromosome of ESCs and PSCs in the IVF, SCNT, and SCNT-RS-1 groups. Comparison of loss and gained regions between the IVF vs. SCNT, IVF vs. SCNT-RS-1, SCNT vs. SCNT-RS-1 groups. Bars indicate the losses and gains of the genes in the chromosome.

| **Table S1. List of the most significant differentially expressed genes (DEGs) in 2-cell stages of the RS-1 treated group (SCNT+RS-1) compared to those of the Kdm4a injected group (SCNT+K)** | | | | | |
| --- | --- | --- | --- | --- | --- |
| **Related function** | **Gene Symbol** | **Gene ID** | **Gene Name** | **Regulation** | **Reference** |
| **Cell survival and**  **Tissue regeneration** | Acap3 | NM_207223.1 | ArfGAP with coiled-coil, ankyrin repeat and PH domains 3 | Up | [^1^](#_ENREF_1) |
|  | Faiml | NM_001244762.1 | Fas apoptotic inhibitory molecule like | Up | [^2^](#_ENREF_2) |
|  | Gipc1 | NM_018771.3 | GIPC PDZ domain containing family, member 1 | Up | [^3^](#_ENREF_3) |
|  | Lmx1a | NM_033652.5 | LIM homeobox transcription factor 1 alpha | Up | [^4^](#_ENREF_4) |
|  | Tnfrsf12a | NM_001161746.1 | tumor necrosis factor receptor superfamily, member 12a | Up | [^5^](#_ENREF_5) |
| **Immune response** | Chit1 | NM_001284524.1 | chitinase 1 (chitotriosidase) | Up | [^6^](#_ENREF_6) |
|  | Ifng | NM_008337.4 | interferon gamma | Up | [^7^](#_ENREF_7) |
|  | Lat2 | NM_020044.3 | linker for activation of T cells family, member 2 | Up | [^8^](#_ENREF_8)^,^[^9^](#_ENREF_9) |
| **Maintenance of pluripotency and self-renewal** | Bcl3 | NM_033601.3 | B cell leukemia/lymphoma 3 | Up | [^10^](#_ENREF_10) |
| **Cell cycle progression** | Bop1 | NM_013481.2 | block of proliferation 1 | Up | [^11^](#_ENREF_11) |
| **DNA repair and genome stabilization** | Fanca | NM_016925.3 | Fanconi anemia, complementation group A | Up | [^12^](#_ENREF_12) |
| **Tumor suppressor** | Gpr161 | NM_001081126.2 | G protein-coupled receptor 161 | Up | [^13^](#_ENREF_13) |
| **mitochondrial metabolism** | P2ry1 | NM_001282016.1 | purinergic receptor P2Y, G-protein coupled 1 | Up | [^14^](#_ENREF_14) |
| **Germ cell proliferation** | Slc22a20 | NM_198650.2 | solute carrier family 22 (organic anion transporter), member 20 | Up | [^15^](#_ENREF_15) |
| **Calcium oscillation** | Sva | NM_009299.2 | seminal vesicle antigen | Up | [^16^](#_ENREF_16) |
| **organize the Golgi architecture** | Yif1b | NM_001110201.1 | Yip1 interacting factor homolog B (S. cerevisiae) | Up | [^17^](#_ENREF_17) |
| **maintain the endothelial homeostasis** | Zc3h12a | NM_153159.2 | zinc finger CCCH type containing 12A | Up | [^18^](#_ENREF_18) |
| **spermatogenesis** | Xlr5c | NM_031493.1 | X-linked lymphocyte-regulated 5C | Down | [^19^](#_ENREF_19) |
| **Unknown** | 0610040B10Rik | NR_027874.1 | RIKEN cDNA 0610040B10 gene | Up |  |
|  | 2010001A14Rik | NR_131042.1 | RIKEN cDNA 2010001A14 gene | Up |  |
|  | 2810428I15Rik | NM_001357209.1 | required for excision 1-B domain containing | Up |  |
|  | 4930430J02Rik | NR_040729.1 | RIKEN cDNA 4930430J02 gene | Up |  |
|  | 4931409K22Rik | NM_177676.6 | IQ motif containing with AAA domain 1 like | Up |  |
|  | AA413626 | NR_102683.1 | expressed sequence AA413626 | Up |  |
|  | AY761185 | NM_001012640.2 | cDNA sequence AY761185 | Up |  |
|  | B3galt4 | NM_019420.2 | UDP-Gal:betaGlcNAc beta 1,3-galactosyltransferase, polypeptide 4 | Up |  |
|  | BC021614 | NM_001362043.1 | glutathione S-transferase pi 3 | Up |  |
|  | Fam109a | NM_001359949.1 | PH domain containing endocytic trafficking adaptor 1 | Up |  |
|  | Ficd | NM_001010825.3 | FIC domain containing | Up |  |
|  | Gm10354 | NM_001281514.1 | predicted gene 10354 | Up |  |
|  | Gm11544 | NM_001205037.1 | predicted gene 11544 | Up |  |
|  | Gm16381 | NM_001166062.2 | predicted gene 16381 | Up |  |
|  | Gm4827 | NR_045935.1 | predicted gene 4827 | Up |  |
|  | Klhl11 | NM_172565.2 | kelch-like 11 | Up |  |
|  | LOC100502896 | NM_001277512.1 | predicted gene 11238 | Up |  |
|  | Mvk | NM_001306205.1 | mevalonate kinase | Up |  |
|  | Pdgfrl | NM_026840.3 | platelet-derived growth factor receptor-like | Up |  |
|  | Ppp1r2-ps7 | NR_033731.1 | protein phosphatase 1, regulatory (inhibitor) subunit 2, pseudogene 7 | Up |  |
|  | Prkag2os1 | NR_040684.1 | protein kinase, AMP-activated, gamma 2 non-catalytic subunit, opposite strand 1 | Up |  |
|  | Ptrh1 | NM_178595.3 | peptidyl-tRNA hydrolase 1 homolog | Up |  |
|  | Sfi1 | NM_001363222.1 | Sfi1 homolog, spindle assembly associated (yeast) | Up |  |
|  | Slc35e3 | NM_029875.2 | solute carrier family 35, member E3 | Up |  |
|  | Snora78 | NR_028515.1 | small nucleolar RNA, H/ACA box 7 | Up |  |
|  | Speer4cos | NR_001585.3 | spermatogenesis associated glutamate (E)-rich protein 4C, opposite strand transcript | Up |  |
|  | Vmn1r88 | NM_001167537.1 | vomeronasal 1 receptor, 88 | Up |  |
|  | Zcchc13 | NM_029158.2 | zinc finger, CCHC domain containing 13 | Up |  |
|  | Rnu12 | NR_004432.2 | RNA U12, small nuclear | Down |  |
|  | Gm4832 | NM_001190356.2 | predicted gene 4832 | Down |  |

**SUPPLEMENTAL REFERENCES**

1. Miura Y, Kanaho Y. ACAP3, the GTPase-activating protein specific to the small GTPase Arf6, regulates neuronal migration in the developing cerebral cortex. *Biochemical and biophysical research communications.* 2017;493(2):1089-1094.

2. Huo J, Xu S, Lin B, Chng WJ, Lam KP. Fas apoptosis inhibitory molecule is upregulated by IGF-1 signaling and modulates Akt activation and IRF4 expression in multiple myeloma. *Leukemia.* 2013;27(5):1165-1171.

3. La Torre A, Hoshino A, Cavanaugh C, Ware CB, Reh TA. The GIPC1-Akt1 Pathway Is Required for the Specification of the Eye Field in Mouse Embryonic Stem Cells. *Stem cells.* 2015;33(9):2674-2685.

4. Doucet-Beaupre H, Gilbert C, Profes MS, et al. Lmx1a and Lmx1b regulate mitochondrial functions and survival of adult midbrain dopaminergic neurons. *Proceedings of the National Academy of Sciences of the United States of America.* 2016;113(30):E4387-4396.

5. Girgenrath M, Weng S, Kostek CA, et al. TWEAK, via its receptor Fn14, is a novel regulator of mesenchymal progenitor cells and skeletal muscle regeneration. *The EMBO journal.* 2006;25(24):5826-5839.

6. Kitamoto S, Egashira K, Ichiki T, et al. Chitinase inhibition promotes atherosclerosis in hyperlipidemic mice. *The American journal of pathology.* 2013;183(1):313-325.

7. Lai HC, Chang CJ, Lin CS, et al. NK Cell-Derived IFN-gamma Protects against Nontuberculous Mycobacterial Lung Infection. *Journal of immunology.* 2018;201(5):1478-1490.

8. Zhu M, Fuller DM, Ou-Yang CW, Sullivan SA, Zhang W. Tyrosine phosphorylation-independent regulation of lipopolysaccharide-mediated response by the transmembrane adaptor protein LAB. *Journal of immunology.* 2012;188(6):2733-2741.

9. Yamasaki S, Ishikawa E, Sakuma M, et al. LAT and NTAL mediate immunoglobulin E-induced sustained extracellular signal-regulated kinase activation critical for mast cell survival. *Molecular and cellular biology.* 2007;27(12):4406-4415.

10. Kang S, Yun J, Kim DY, et al. Adequate concentration of B cell leukemia/lymphoma 3 (Bcl3) is required for pluripotency and self-renewal of mouse embryonic stem cells via downregulation of Nanog transcription. *BMB reports.* 2018;51(2):92-97.

11. Strezoska Z, Pestov DG, Lau LF. Functional inactivation of the mouse nucleolar protein Bop1 inhibits multiple steps in pre-rRNA processing and blocks cell cycle progression. *The Journal of biological chemistry.* 2002;277(33):29617-29625.

12. Yang YG, Herceg Z, Nakanishi K, et al. The Fanconi anemia group A protein modulates homologous repair of DNA double-strand breaks in mammalian cells. *Carcinogenesis.* 2005;26(10):1731-1740.

13. Shimada IS, Hwang SH, Somatilaka BN, et al. Basal Suppression of the Sonic Hedgehog Pathway by the G-Protein-Coupled Receptor Gpr161 Restricts Medulloblastoma Pathogenesis. *Cell Rep.* 2018;22(5):1169-1184.

14. Zheng W, Watts LT, Holstein DM, et al. Purinergic receptor stimulation reduces cytotoxic edema and brain infarcts in mouse induced by photothrombosis by energizing glial mitochondria. *PLoS One.* 2010;5(12):e14401.

15. Schnabolk GW, Gupta B, Mulgaonkar A, Kulkarni M, Sweet DH. Organic anion transporter 6 (Slc22a20) specificity and Sertoli cell-specific expression provide new insight on potential endogenous roles. *J Pharmacol Exp Ther.* 2010;334(3):927-935.

16. Lu SH, Yen YK, Ling TY, et al. Capacitation suppression by mouse seminal vesicle autoantigen involves a decrease in plasma membrane Ca2+-ATPase (PMCA)-mediated intracellular calcium. *J Cell Biochem.* 2010;111(5):1188-1198.

17. Alterio J, Masson J, Diaz J, et al. Yif1B Is Involved in the Anterograde Traffic Pathway and the Golgi Architecture. *Traffic.* 2015;16(9):978-993.

18. Jin ZL, Shen XH, Shuang L, Kwon JW, Seong MJ, Kim NH. Inhibition of DNA repair protein RAD51 affects porcine preimplantation embryo development. *Reproduction.* 2019;157(3):223-234.

19. Zhuang XJ, Tang WH, Liu CY, et al. Identification and Characterization of Xlr5c as a Novel Nuclear Localization Protein in Mouse Germ Cells. *PLoS One.* 2015;10(6):e0130087.
